# A case control study of premorbid and currently reported physical activity levels in chronic fatigue syndrome

**DOI:** 10.1186/1471-244X-6-53

**Published:** 2006-11-13

**Authors:** Wayne R Smith, Peter D White, Dedra Buchwald

**Affiliations:** 1 Departments of Psychiatry and Behavioral Sciences (W.S) and Medicine (D.B), University of Washington, Seattle, USA; 2 Centre for Psychiatry, Wolfson Institute of Preventive Medicine, Barts and the London, Queen Mary University of London, UK (P.D.W)

## Abstract

**Background:**

Patients with chronic fatigue syndrome typically report high levels of physical activity before becoming ill. Few studies have examined premorbid and current activity levels in chronically fatigued patients.

**Methods:**

In a case-control study, 33 patients with chronic, unexplained, disabling fatigue attending a university-based clinic specializing in fatigue were compared to 33 healthy, age- and sex-matched controls. Patients rated their activity levels before their illness and currently, using scales designed for this purpose. Controls reported their level of activity of 2 years previously and currently. Chi-square analyses, Student's t tests, and Wilcoxon signed rank tests were used in pair matched analyses.

**Results:**

Compared to healthy controls, patients with chronic, unexplained fatigue rated themselves as more active before their illness (*p *≤ 0.001) and less active currently (*p *≤ 0.001). The patients also reported they currently stood or walked less than the controls (median [inter-quartile range] = 4 [2-5] versus 9 [7.5–12] hours, *p *≤ 0.001), and spent more time reclining (median [inter-quartile range] = 12 [10-16] versus 8 [8–9.5] hours, *p *≤ 0.001). These differences remained significant for the subset of patients who met strict criteria for chronic fatigue syndrome or fibromyalgia.

**Conclusion:**

Patients with chronic, unexplained, disabling fatigue reported being more active before becoming ill than healthy controls. This finding could be explained by greater premorbid activity levels that could predispose to illness, or by an overestimation of previous activity. Either possibility could influence patients' perceptions of their current activity levels and their judgments of recovery. Perceived activity should be addressed as part of management of the illness.

## Background

Chronic, unexplained, disabling fatigue is reported by up to 2.6 per cent of patients seeking health care [1]. Chronic fatigue syndrome (CFS) is a more narrowly defined illness with prominent fatigue and at least 4 associated symptoms [2]. Many CFS patients also suffer from fibromyalgia [3], a disabling condition characterized by chronic widespread pain, fatigue, and sleep disturbances [4]. Individuals with CFS often perceive physical activity as more of an effort than healthy people [5], underestimate their cognitive and physical abilities [5-9], and are more aware of, and focused on, their internal physiological state [7,9,10]. Furthermore, CFS patients aspire to greater activity levels [6], and rate themselves as having been significantly more active [6] and "action-prone" before they became ill, than do control subjects [11]; "action-proneness" also increases with treatment [12]. The high levels of physical activity reported by patients have been corroborated by their spouses, partners, or parents [13].

The etiology of chronic, unexplained fatigue, CFS, and fibromyalgia remains unclear [7], but recent work suggests that these disorders may involve enhanced interoception [14,15]. Interoception is the perception of internal sensory phenomena, especially visceral perceptions [16]. The biological underpinnings of interoception are only now being explored, but intriguing neuroimaging studies suggest that a discrete interoceptive cortex, the anterior insula, modulates this phenomenon [17]. If interoception were altered in chronically fatigued individuals, this could affect their internal perception and, consequently, their perceived ability and capacity.

To replicate previous findings and extend them to well-defined subgroups, we conducted a clinic-based, case-control study of fatigued patients, a large majority of whom suffered from CFS and/or fibromyalgia. We compared self-reported premorbid and current activity levels of the patients and their healthy controls to address the following questions: 1) Do patients with chronic, unexplained, disabling fatigue, including the subsets with CFS and fibromyalgia, perceive their premorbid activity levels as higher than the current activity levels perceived by healthy matched controls? 2) Do patients with chronic, unexplained, disabling fatigue, including the subsets with CFS and fibromyalgia, perceive their current activity levels as lower than those perceived by healthy matched controls?

## Methods

### Subjects and Setting

Patients were adults evaluated in a university-based referral clinic for chronic fatigue and pain. The clinic accepts both self- and physician-referred patients; individuals were not required to meet case definitions for either CFS or fibromyalgia to be evaluated. Patients underwent an intake evaluation that included a standardized physical examination, medical history, questionnaire on past and current symptoms, screening laboratory tests, and a structured psychiatric interview. A lay interviewer administered the National Institutes of Mental Health Diagnostic Interview Schedule Version III-A [18], a structured interview that assigns current and lifetime psychiatric diagnoses based on criteria established in the *Diagnostic and Statistical Manual of Mental Disorders, 3*^*rd*^*ed. (Revised) *[19] for somatization disorder, panic disorder, generalized anxiety disorder, major depression, dysthymia, and alcohol abuse/dependence. CFS and fibromyalgia were diagnosed according to the guidelines of the Centers for Disease Control and Prevention [20] and the American College of Rheumatology [4], respectively. Those who did not meet the CFS case definition met the criteria for idiopathic chronic fatigue [20]. Fibromyalgia often co-occurred with idiopathic, chronic fatigue and CFS, as previously described [3].

To recruit controls, we asked patients the following: "We would like to compare the activity levels of people with chronic fatigue to healthy people. If you have a healthy friend who would agree to complete [questions about activity] only, please fill out the information below. If possible, pick someone who is similar to you in terms of sex and age." In this way, 33 controls were recruited. The University of Washington Human Subjects Office reviewed and approved all clinic procedures and consent forms.

### Measures

The questionnaire for this study was mailed along with an annual clinic newsletter. Non-responders were mailed the questionnaire a second time, followed by an attempt to gather the information by telephone. The questionnaire contained 4 items specifically asking about levels of activity [see Additional file 1]. Patients were asked to rate their typical levels of activity prior to becoming chronically fatigued and compare it to an average healthy person by using a 10-point scale (1 = extremely low to 10 = extremely high). Using the same scale, patients were also asked to rate their typical level of activity during the previous 7 days as compared to that of an average healthy person [see Additional file 1]. The other 2 items inquired about activity during the previous 24 hours. One instructed patients to estimate how many hours they had spent standing or walking, sitting, or reclining or lying down. The other asked if this level of activity was higher, lower, or average compared to their recent activity levels.

For the healthy controls, the first of the 4 activity questions was revised to ask about their activity as of 2 years ago compared to the average healthy person. The two-year recall period was chosen because the mean fatigue duration reported by patients evaluated in the clinic was nearly 2 years. The remaining 3 questions were identical to those given to patients. No other information was collected from the control subjects.

### Statistical Analyses

Respondents were classified as patients or their friends (i.e., controls). To examine between-group differences, chi-square analyses were used for dichotomous variables and interval variables were compared by using Student's t test when data were normally distributed, and by using Wilcoxon signed rank tests when data were not normally distributed, always using pair-related comparisons.

## Results

### Demographic and Clinical Characteristics

Overall, 462 of 678 (68%) consecutively evaluated clinic patients completed the questionnaire. Of these 462, 44 patients had a friend who completed the 4 activity items; these 44 patients and their friends constituted the study sample. The 44 patients tended to be older than the larger clinic population (43.6 vs. 39.6 years, *p *= 0.02), but did not differ in gender (84% vs. 74% female), marital status (50% vs. 50% married), duration of fatigue (5.9 vs. 5.2 years), or proportion diagnosed with CFS (57% vs. 56%) or fibromyalgia (21% vs. 23%). Likewise, our study sample had rates similar to those of all clinic patients for lifetime diagnoses of alcohol abuse (14% vs. 16%), major depression (69% vs. 65%), dysthymic disorder (26% vs. 22%), generalized anxiety disorder (17% vs. 24%), panic disorder (26% vs. 19%), and somatization disorder (29% vs. 21%). However, they tended to be diagnosed more often with melancholic depression (11% vs. 5%, *p *= 0.02).

Of the 44 fatigued clinic patients, we limited our analysis to the 33 who met criteria for CFS, idiopathic chronic fatigue, and/or fibromyalgia. The other 11 patients were excluded for medical or psychiatric conditions that could explain their fatigue [21]. The demographic and clinical characteristics of the 33 patients and their 33 matched controls are presented in Table 1. Patients and controls did not differ in age (*p *= 0.22) or sex (*p *= 0.50). Of the 33 patients with chronic, unexplained, debilitating fatigue, 25 (76%) met the criteria for CFS, 7 (21%) met the criteria for idiopathic chronic fatigue, and 9 (27%) met criteria for fibromyalgia. Only 1 had fibromyalgia alone, without CFS or idiopathic chronic fatigue.

**Table 1:** Demographic and clinical characteristics of patients and healthy control subjects

**Characteristic**	**Patients**	**Controls**
***Demographic***		
Number	33	33
Age, *mean years (SD)*	45.6 (11.8)	44.1 (13.1)
Female, *n (%)*	26 (79)	28 (85)
***Clinical***		
CFS criteria met, *n (%)*	25 (76)	---
Idiopathic chronic fatigue criteria met *n (%)*	7 (21)	
Fibromyalgia criteria met ^1^, *n (%)*	9 (27)	---

^1 ^1 patient had fibromyalgia, but neither CFS nor idiopathic chronic fatigue.

Table 2 illustrates that, compared with their matched controls, the 33 patients rated themselves as more active before the onset of illness (Z score -3.05, *p *= 0.002) and less active currently (Z score -4.72, *p *< 0.001). More specifically, in the previous 24 hours, patients reported standing or walking 5 hours less than their healthy friends (Z score -4.39, *p *< 0.001) and lying or reclining 4 hours more (Z score -4.29, *p *< 0.001), with no differences in the time spent sitting (Z score -0.86, *p *= 0.39). For both patients and controls, the activity reported during the previous 24 hours represented a typical day (Z score -1.12, *p *= 0.26).

**Table 2:** Previous and current activity levels of patients and healthy control subjects

**Characteristic**	**Patients**	**Controls**
**Previous activity level**^1,2^, *median (IQR) *^3^	9 (8 – 9.5)	8 (6.5 – 8.5)*
**Previous activity level, ***n (%)*		
Level 1	0	0
Level 2	0	0
Level 3	0	1 (3)
Level 4	1 (3)	1 (3)
Level 5	0	2 (6)
Level 6	1 (3)	4 (12)
Level 7	3 (9)	5 (15)
Level 8	8 (24)	12 (36)
Level 9	12 (36)	5 (15)
Level 10	8 (24)	3 (9)
**Last week's activity**^2^, *median (IQR) *^3^	3 (2 – 5)	8 (6 – 8) ^†^
**Last week's activity**, *n (%)*		
Level 1	5 (15)	0
Level 2	6 (18)	0
Level 3	7 (21)	1 (3)
Level 4	4 (12)	2 (6)
Level 5	5 (15)	3 (9)
Level 6	3 (9)	4 (12)
Level 7	1 (3)	5 (15)
Level 8	2 (6)	11 (33)
Level 9	0	4 (12)
Level 10	0	3 (9)
**Last 24 hours' activities, ***median hours (IQR)*^3^		
Standing or walking	4 (2 – 5)	9 (7.5 – 12) ^†^
Sitting	6 (5 – 9)	6 (4 – 8)
Reclining or lying down	12 (10 – 16)	8 (8 – 9.5) ^†^
**Activity Level in Last 24 Hours**, *%*^4^		
Higher than recently	6	24
Lower than recently	45	27
Average	49	48

^1 ^premorbid activity level for patients, activity level 2 years ago for friends; ^2 ^rated from 1 (extremely low) to 10 (extremely high); ^3 ^interquartile range; ^4 ^compared to recent levels.

*p ≤ 0.01; ^† ^*p *≤ 0.001

Lastly, we considered premorbid and current activity levels in the CFS and fibromyalgia subgroups. These results are not displayed in the table. The median rating (and interquartile range) for premorbid activity level was 9 (8 – 9.5) among the 25 CFS patients versus 8 (7 – 9) among controls (Z = -2.18, *p *= 0.03). Among CFS patients, the median rating (and interquartile range) for current activity was 3 (2 – 5) versus 8 (6.5 – 8.5) among controls (Z = -4.23, *p *≤ 0.001). In the 9 patients with fibromyalgia, the median rating (and interquartile range) for premorbid activity was 9 (8 – 9.5) versus 8 (7 – 8.5) among controls (Z = -2.46, *p *= 0.01), and the current activity was lower: 4 (1.5 – 4.5) versus 7 (6 – 8) (Z = -2.53, *p *= 0.01).

## Discussions and Conclusion

We have confirmed the previous finding that patients with CFS report higher levels of premorbid activity than do healthy control subjects, and we have extended this finding to include patients with chronic, unexplained fatigue and fibromyalgia. As expected, both CFS and fibromyalgia patients reported less current activity than healthy controls, but small sample sizes and Bonferroni's corrections resulted in borderline significance for some of the sub-group comparisons. Our findings are congruent with those of 3 retrospective studies reporting that CFS patients perceived themselves as more active before their illness began than healthy controls [6,11,21]. In contrast, the only prospective cohort study of risk factors for CFS found that sedentary behavior at 10 years of age doubled the risk of self-reported CFS in adulthood [22]. Since no prospective data exist on the actual pre-morbid activity levels of individuals who later suffer a fatiguing illness, it is unclear whether our data reflect pre-morbid excessive activity, or alternatively, illness-related alterations in the perception or recall of premorbid activity.

One of the "altered-perception" hypotheses involves sensitized interoception. CFS and fibromyalgia have been postulated to be central nervous system hypersensitivity disorders, characterized by enhanced or sensitized interoception [15,23,24]. A central nervous system hypersensitivity disorder would be consistent with patients' reports of disturbed cognition and concentration and sensitivity to exercise, chemicals, and odors [5,7-9,25]. Support for this explanation comes from investigations that have described discrepancies between subjectively reported impairments and objective measures of activity [26,27], effort with exercise [5,28,29], sleep quantity and quality [30], and cognitive symptoms and cognitive abilities [8,9,25,29].

Of interest, humans appear to have a distinct cortical image of homeostatic afferent activity that reflects all aspects of the physiological condition of all tissues of the body. This interoceptive system is linked to the autonomic nervous system, but is distinct from the exteroceptive system that reflects somatic motor activity. The primary interoceptive representation in the anterior insula cortex engenders distinct bodily sensations, including pain, temperature, itch, sensual touch, muscular and visceral sensations, and vasomotor activity [31]. Neuroimaging studies relevant to interoception and the feeling of self are rapidly accumulating and underscore the biological underpinnings of this phenomenon [16,17].

Lastly, perception and consequent ability, as well as external agents, may influence patients' beliefs. For example, when benign chemicals were given blindly to people with CFS and multiple chemical sensitivities, individuals who believed they were getting the chemical performed poorly on cognitive tests, whereas cognitive function did not differ between those who received the chemicals and those who did not [25]. In another investigation, fatigued people and CFS patients had greater doubts about actions and more concerns over mistakes than controls [32,33]. Such self-critical personality traits might diminish judgments of current abilities and exaggerate previous abilities.

This study has several limitations. First, our sample was drawn from consecutive patients evaluated at a referral clinic. Because patients who present to specialty clinics probably differ from community samples or those drawn from primary care clinics on important variables [34], our results may not be generalizable to other settings or other fatigued patients. Second, because our measures were obtained at only one time point, we do not know if these activity levels represent sustained or transitory levels. Similarly, to minimize the burden on the control subjects, we did not ask them to provide detailed demographic information. Third, our measures of activity were brief and not validated against actigraphy or even the report of the friends, and therefore are subject to the usual biases associated with self-reported health-related behavior, including the propensity to over-value pre-illness functioning. Fourth, we could not verify objectively that the selected friend was actually healthy, yet the presence of chronic conditions would decrease the differences between groups and thus render our findings less significant. Lastly, only a small fraction of the total clinic population obtained data from a friend. The small sample that we examined, however, was similar in most respects to the larger clinic population.

In conclusion, we have replicated previous work showing that CFS patients report greater premorbid activity levels than healthy controls, and we have extended these observations to more rigorously defined fatiguing conditions. These findings suggest that at this point clinical management should address both perceptions of symptoms as well as actual activity levels. Cognitive behavior therapy and graded exercise programs are designed to change both dimensions, and have been shown to be efficacious treatments for the majority of adult, ambulant CFS patients [35,36]. Other innovative interventions that can be easily implemented and are acceptable to patients are also needed.

## List of Abbreviations

CFS = chronic fatigue syndrome

SD = standard deviation

## Competing interests

The author(s) declare that they have no competing interests.

## Authors' contributions

DB designed and implemented the intervention, and edited the manuscript. WRS managed data flow, undertook preliminary analyses, and edited and revised the manuscript. PDW undertook final analyses and wrote majority of the manuscript. All authors read and approved the final manuscript.

## Pre-publication history

The pre-publication history for this paper can be accessed here:



## Supplementary Material

Additional File 1 Chronic Fatigue Activity Questionnaires. Fours questions completed by patients followed by the corresponding four questions completed by the matched controls. Click here for file
