# Supplementary material for: A case control study of premorbid and currently reported physical activity levels in chronic fatigue syndrome
Source: BMC Psychiatry. 2006 Nov 13;6:53. doi: 10.1186/1471-244X-6-53 (PMC1647270; doi:10.1186/1471-244X-6-53)
Supplement: Additional File 1 — Chronic Fatigue Activity Questionnaires. Fours questions completed by patients followed by the corresponding four questions completed by the matched controls. [file 1471-244X-6-53-S1.doc]

# Chronic Fatigue Activity Questionnaire, for PATIENTS

# (last 3 questions of longer questionnaire)

1. Think back to your activities before you became ill. Please rate your typical level of daily activity as it was prior to the time you became chronically fatigued. Use the scale below to compare your prior level of activity to that of an “average” healthy person.

My previous activity level was:

|----------|----------|----------|----------|----------|----------|----------|----------|----------|

extremely low average high extremely

low high

1. Please rate your typical level of activity during the last 7 days. Use the scale below to compare your current level of activity to that of an “average” healthy person.

My activity level during the past week was:

|----------|----------|----------|----------|----------|----------|----------|----------|----------|

extremely low average high extremely

low high

1. Estimate how much time you spent during the last 24 hours in each of the three types of activities listed below (the total number of hours should equal 24).

Standing or walking ______hours

Sitting ______hours

Reclining or lying down ______hours

Was your activity during the last 24 hours: (check one)

___higher than recently

___lower than recently

___average

# Chronic Fatigue Activity Questionnaire, for CONTROLS

We are interested in the activity levels of people with chronic fatigue and those who do not have any type of chronic illness. Please complete the questions below and return the questionnaire to us.

1.Name of the person who gave this questionnaire to you________________

2. Your age ____________

3. Your sex M F

4. List any type of current health problems you have which affect your level of activity_____________________________________________________

1. Think back to your activities about two years ago. Please rate your typical level of daily activity as it was two years ago. Use the scale below to compare your prior level of activity to that of an “average” healthy person.

My previous activity level was:

|----------|----------|----------|----------|----------|----------|----------|----------|----------|

extremely low average high extremely

low high

1. Please rate your typical level of activity during the last 7 days. Use the scale below to compare your prior level of activity to that of an “average” healthy person.

My activity level during the past week was:

|----------|----------|----------|----------|----------|----------|----------|----------|----------|

extremely low average high extremely

low high

1. Estimate how much time you spent during the last 24 hours in each of the three types of activities listed below (the total number of hours should equal 24).

Standing or walking ______hours

Sitting ______hours

Reclining or lying down ______hours

Was your activity during the last 24 hours a) higher, b) lower or c) average for you recently? (circle one)

THANK YOU FOR YOUR HELP
